# Method for detection of cerebral blood flow in neurointensive care using longitudinal arterial spin labeling MRI

**DOI:** 10.1371/journal.pone.0314056

**Published:** 2024-11-19

**Authors:** Sofie Tapper, Anders Tisell, Jan Hillman, Karin Wårdell

**Affiliations:** 1 Department of Biomedical Engineering, Linköping University, Linköping, Sweden; 2 Department of Medical Radiation Physics, Linköping University Hospital, Linköping, Sweden; 3 Department of Health, Medicine and Caring Sciences, Linköping University, Linköping, Sweden; 4 Department of Neurosurgery, Linköping University Hospital, Linköping, Sweden; 5 Department of Biomedical and Clinical Sciences, Linköping University, Linköping, Sweden; Universitatsklinikum Regensburg, GERMANY

## Abstract

Cerebral blood flow (CBF) is carefully monitored in the Neurointensive Care Unit (NICU) to prevent secondary brain insults in patients who have suffered subarachnoid hemorrhage. Including absolute MRI measurements of CBF in the NICU monitoring protocol could add valuable information and potentially improve patient outcomes. This is particularly feasible at Linköping University Hospital, which uniquely has an MRI scanner located in the NICU, enabling longitudinal CBF measurements while eliminating medical transportation risks. Arterial spin labeling is a subtraction-based MRI technique that can measure CBF globally in the brain without the use of contrast agents, and thus is suitable for repeated measurements over short time periods. Therefore, this work aims to develop and implement a methodological workflow for the acquisition, analysis, absolute quantification, and visualization of longitudinal arterial spin labeling MRI measurements acquired in the clinical NICU setting. At this initial stage, the workflow was implemented and tested using acquired test-retest data and longitudinal data from two healthy participants. Subsequently, the workflow was tested in clinical practice on an intubated and ventilated patient monitored in the NICU after suffering a subarachnoid hemorrhage. To ensure accurate day-to-day comparisons between the repeated measurements, the selection of processing and analysis methods aimed to obtain CBF maps in absolute units of ml/min/100g. These CBF maps were quantified using both the FMRIB Software Library and an openly available flow territory atlas. The test-retest data showed small variations (4.4 ml/min/100g between sessions), and the longitudinal measurement resulted in low CBF variability over 12 days. Despite the greater complexity of clinical data, the quantification and chosen visualization tools proved helpful in interpreting the results. In conclusion, this workflow including repeated MRI measurements could help detect changes in CBF between different measurement days and complement other conventional monitoring techniques in the NICU.

## Introduction

In the Neurointensive Care Unit (NICU), patients are carefully monitored after suffering from a traumatic brain injury (TBI) or brain hemorrhages, such as subarachnoid hemorrhage (SAH). These primary injuries can trigger pathophysiological events that may lead to delayed cerebral ischemia (DCI) and subsequent brain infarction [1]. One reason for DCI is cerebral vasospasm, a prolonged but reversible narrowing of cerebral arteries that can occur in both TBI and SAH cases. Prophylactic administration of the calcium antagonist nimodipine offers protection against vasospasm. However, in severe and progressive cases, the most effective treatments are endovascular administration of nimodipine or balloon angioplasty [2]. Vasospasm complications typically arise without clear warnings, usually between 3 to 14 days after the primary injury [3]. Early detection is crucial for improving patient outcomes, which is why patients are carefully monitored for up to 14 days in the NICU after suffering a cerebral hemorrhage.

The monitoring setup is normally multimodal, incorporating measurements such as intracranial pressure, brain tissue oxygenation, and occasionally brain metabolism using microdialysis [4]. Additionally, changes in the cerebral blood flow (CBF) can also be indicative of vasospasm or DCI. However, measuring CBF in the NICU setting is not straightforward, as the available techniques each have different advantages and disadvantages. At Linköping University Hospital, the conventional monitoring protocol includes daily measurements of cerebral blood flow velocity (CBFV) in the middle cerebral artery (MCA) using transcranial Doppler (TCD) ultrasonography [5]. In cases of SAH, an abnormal CBFV may indicate vasospasm, and subsequent treatment with nimodipine may potentially improve long-term outcomes [6]. Xenon-enhanced Computed Tomography (Xe-CT) is another bedside monitoring technique that has previously been part of the monitoring protocol [7]. During Xe-CT measurements, the patient inhales xenon gas while CT images are acquired using a mobile CT system. The diffusion of the Xenon gas into the tissue reveals the CBF in each area of the brain, measured in the absolute units of ml/min/100g [7]. In contrast to Xe-CT, blood flow measurements using Magnetic Resonance Imaging (MRI) are both non-invasive and free of radiation exposure. Instead of radiation, MRI scanners use magnetic fields and radio waves to interact with hydrogen atoms in the body, producing detailed images. At the Department of Neurosurgery, Linköping University Hospital, a unique setup is available with an MRI scanner located in the NICU. This setup enables long-term observation of monitored patients using MRI-based techniques without exposing the patient to the risks of intra-hospital transfer [8]. However, conventional MRI, such as dynamic susceptibility contrast MR perfusion, requires a contrast agent to measure CBF [9]. Therefore, long-term repeated perfusion measurements are not recommended due to the increased risks associated with repeated gadolinium exposure [10].

Arterial Spin Labeling (ASL) [11–13] is an MRI modality that does not require any contrast agent and is thus suitable for repeated CBF measurements in the NICU setting. The ASL measurement generates both a control image (without labeling) and a label image where inflowing arterial blood water spins have been inverted or saturated. The label image is subtracted from the control, and the resulting signal intensity in this difference image may exclusively be attributed to CBF. There are currently numerous implemented ASL schemes such as Pulsed ASL (PASL) [14–16], pseudo-Continuous ASL (pCASL) [17], and Velocity-Selective ASL (VSASL) [18], with the main difference in how the labeling is being performed. Our clinical MRI scanner in the NICU has a PASL sequence available and approved for clinical use. In PASL, blood water is magnetically inverted as it passes through a labeling slab placed over the arteries in the neck [19]. Using repeated PASL to detect changes in CBF over several days in the NICU highlights the need for comparable CBF maps between measurements, expressed in absolute units, similar to the CBF maps obtained with Xe-CT [20–22]. Therefore, the signal intensity of fully relaxed blood spins is needed for scaling the CBF maps on a voxel-by-voxel basis. One strategy is to use proton density weighted (PDw) images acquired separately during the same measurement session, as recommended in the ASL consensus paper, commonly referred to as the ‘white paper’ [19].

Including absolute MRI measurements of CBF into the NICU monitoring protocol could provide valuable information that may improve patient outcomes. Therefore, the aim of this work was to develop and implement a methodological workflow for the acquisition, analysis, absolute quantification, and visualization of longitudinal PASL measurements performed in the clinical NICU setting. The intention with this workflow was to detect changes in CBF between measurement days, which could indicate that the patient is progressing into cerebral vasospasm or DCI. Initially, two healthy volunteer measurements were performed to implement the workflow, which later was tested in practice on an intubated and ventilated patient monitored in the NICU after suffering an SAH.

## Materials and methods

**Fig 1:**
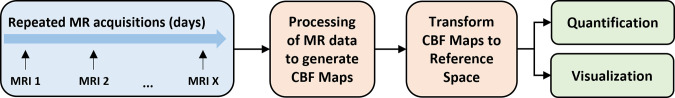
Illustration of the workflow. This workflow outlines the key steps, from MR acquisitions in the NICU to the quantification and visualization of the resulting absolute CBF maps.

This study was designed to investigate the feasibility of using repeated MRI measurements of CBF in the NICU setting. This section details the MR acquisitions, data processing, quantification, and visualization methods, collectively referred to as the implemented workflow. In addition, this section also provides information on the study participants and the procedures used to evaluate the workflow. Fig 1 illustrates the key steps of the implemented workflow, including the MR acquisitions, processing of each dataset to generate absolute CBF maps, transformation of all maps to the same reference space, and the subsequent quantification and visualization.

### MR acquisitions

While the patient is monitored in the NICU, the intention is to perform occasional MRI measurements, which frequency depends on scanner availability and the current condition of the patient. The NICU in Linköping has access to a clinical 3 T MAGNETOM Skyra MR system (Siemens Healthineers, Erlangen, Germany), equipped with a 20-channel head coil for these measurements. The MR protocol parameters remained consistent across all acquisitions, including PASL imaging, Proton Density-weighted (PDw) imaging, and T1-weighted (T1w) imaging, which were performed in each MR protocol. Following an image scout and subsequent measurement planning (with the imaging volume aligned along the AC-PC line), PASL data was acquired using perfusion mode FAIR QII [23]. The acquisition parameters were TE/TI/TI1/TR  =  15.56/1990/700/4600 ms, FOV  =  205 × 205 mm^2^, acquired resolution = 3.25 x 3.20 x 3.00 mm^3^, reconstructed resolution = 1.6 x 1.6 x 3.0 mm^3^, base resolution = 64, bandwidth  =  2694 Hz/px, 3D GRASE readout, EPI factor  =  21, turbo factor = 20, and scan time = 4 min 59 s. Four control-pairs of 40 slices with 3 mm thickness were collected. The labeling slab was fixed in position and consistently followed the imaging volume across all measurements [24]. To scale the CBF maps to absolute units of ml/min/100g, PDw turbo spin echo images were acquired with the following parameters: TE/TR = 8.6/10000 ms, flip angle = 160 deg, and scan time = 2 min 22 s. The geometry was consistent with the geometry used in the acquisition of the PASL data, with a FOV = 205 x 205 mm^2^ and slice thickness of 3 mm. Additionally, high-resolution 3D MPRAGE T1w sagittal images were acquired for image registration and tissue segmentation, using the following parameters: TE/TI/TR = 2.29/900/2300 ms, flip angle = 8 deg, FOV = 240 x 240 mm^2^, slice thickness = 1 mm, voxel size = 0.9 x 0.9 x 1.0 mm^3^, and a scan time of 5 min 21 s.

### Processing of MR data

**Fig 2:**
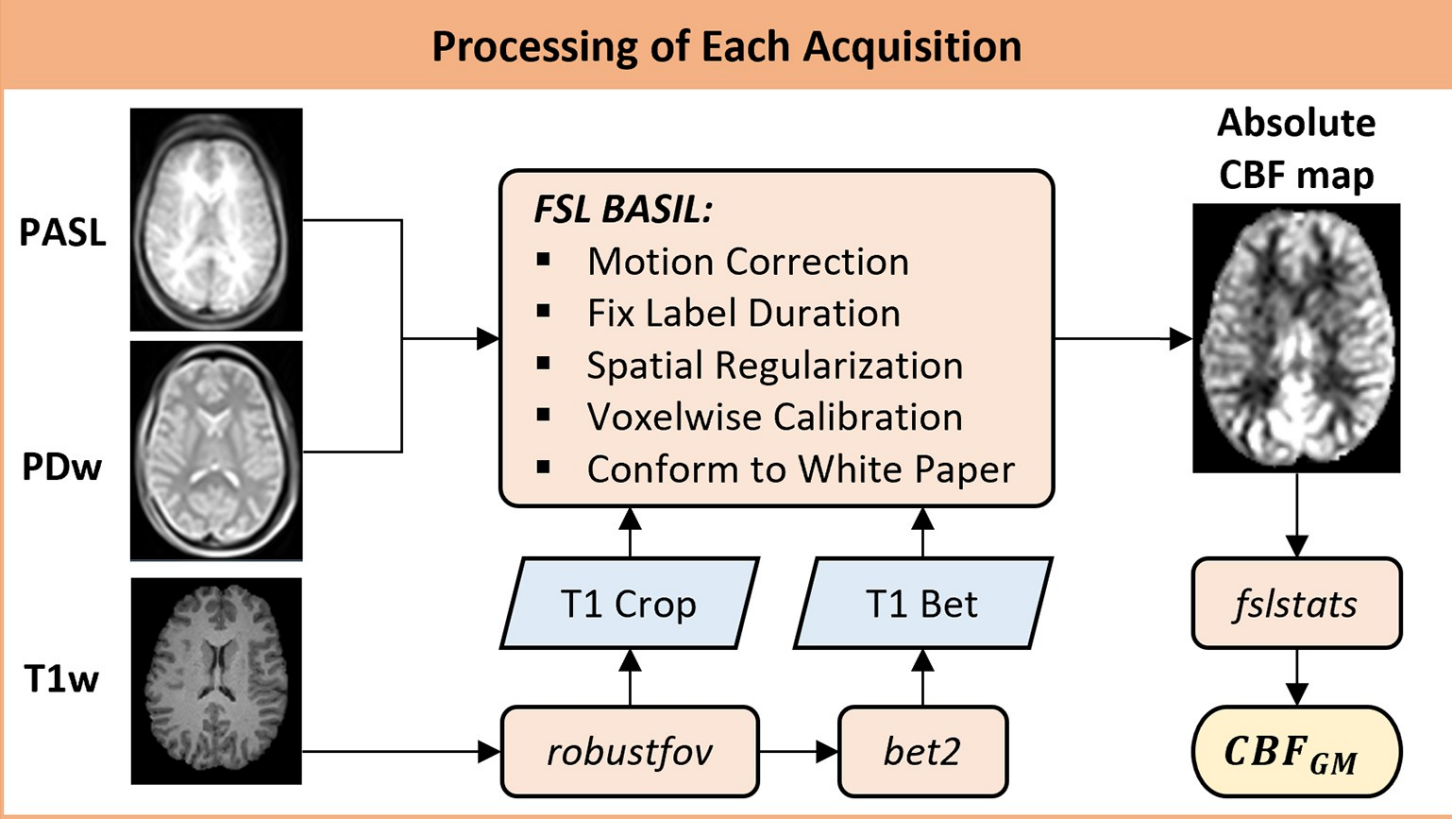
Processing steps performed for each MR acquisition. The MRI protocol involved acquiring PASL, PDw, and T1w images for each measurement. The T1w images were optimally cropped using *robustfov*, followed by brain extraction with *bet2*. PASL data was processed using the FMRIB software library (FSL), adhering to the guidelines outlined in Alsop et al. (2015) [19]. The absolute CBF map was quantified using *fslstats*, resulting in an estimate of the CBF in grey matter (${CBF}_{GM}$).

Each MR dataset was processed according to Fig 2 to generate an absolute CBF map, primarily using functions from the FMRIB Software Library (FSL, v6) [25, 26]. Structural T1w images were optimally cropped using *robustfov* to remove slices containing the neck, ensuring precise center coordinates for accurate brain extraction using *bet2* [27]. The PASL data was processed using the *BASIL GUI* [28], with settings that included calibration using M0 type Proton density long TR (10 s) with a voxelwise approach and calibration gain 10. No distortion correction was applied, and the analysis conformed to the white paper [19]. Additionally, the following options were enabled: adaptive spatial regularization on perfusion, fix label duration, and motion correction. The resulting absolute CBF map remained in native space, and *fslstats* was used to calculate an estimate of the mean CBF in grey matter (${CBF}_{GM}$) based on partial volume estimates (percentage of grey matter (GM), white matter, and CSF) from the T1w images. For all acquisitions, a voxel was considered pure GM if the partial volume estimate was equal to or above 80% GM [29]. Processing the data took about 1 hour per acquisition when performed on a 2.67 GHz Intel Xeon CPU.

### Quantification of CBF maps

**Fig 3:**
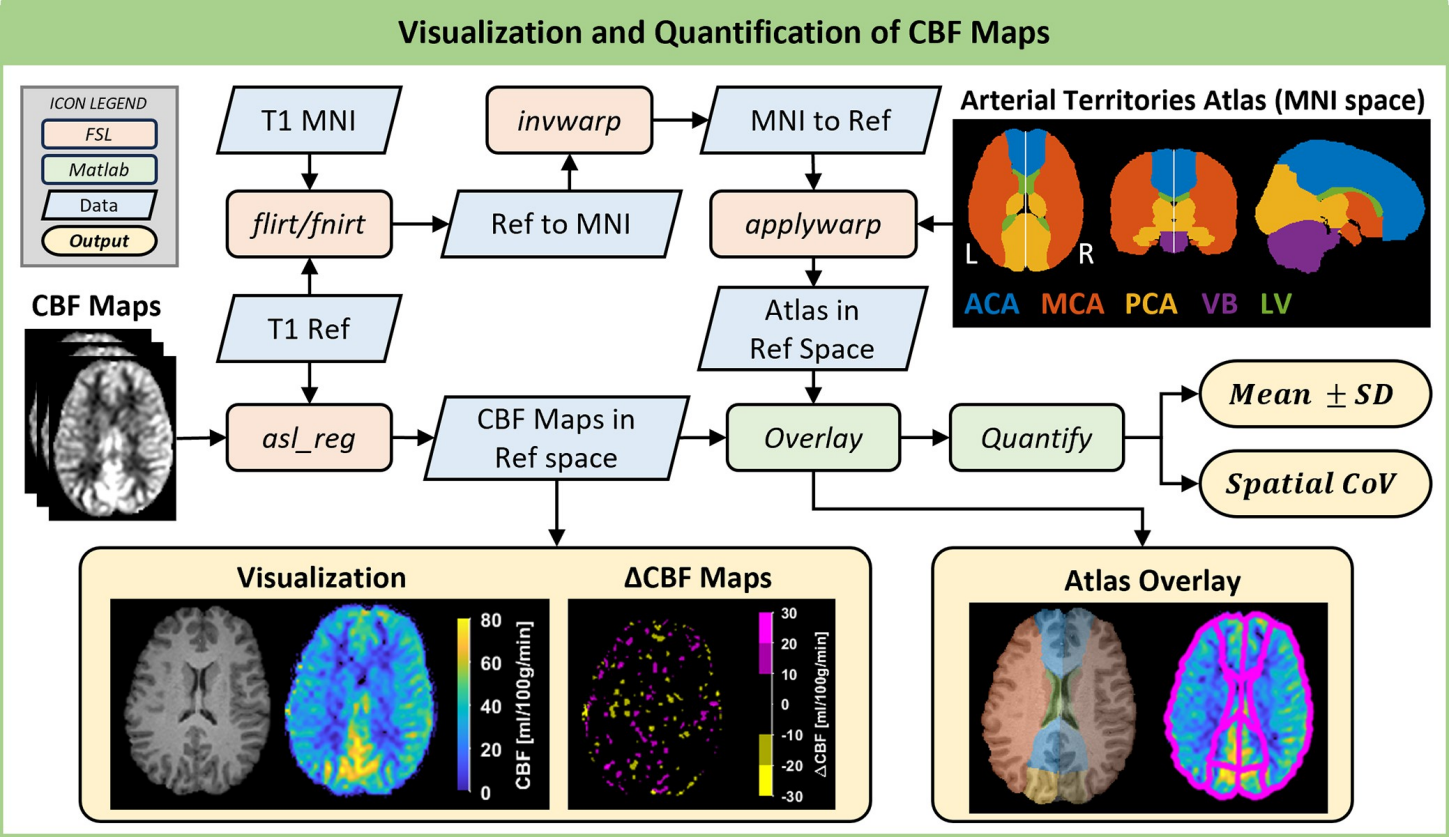
Quantification and visualization of CBF maps. The resulting CBF maps acquired from one participant were transformed to the same reference space (T1 Ref) before visualization and quantification. The visualizations of CBF and ΔCBF maps were created using specially designed color schemes to highlight changes in CBF. The arterial territories atlas was transformed from MNI space to the same reference space (T1 Ref). Subsequently, the mean ± SD and spatial coefficient of variation (CoV) were calculated for each arterial territory. Territories: left and right anterior cerebral artery (ACA), middle cerebral artery (MCA), posterior cerebral artery (PCA), vertebro-basilar artery (VB), and lateral ventricle (LV).

Fig 3 illustrates the steps involved in quantifying and visualizing the CBF maps obtained from a single participant. To facilitate detailed comparisons of CBF maps acquired on different days, it is essential that all resulting absolute CBF maps are aligned within the same spatial framework. A participant-specific reference space was established using one of the structural T1w images. Subsequently, all CBF maps were transformed into this reference space using the FSL function *asl_reg*, which is designed to register low-resolution ASL images to structural images [28]. This transformation adjusted the resolution of the absolute CBF maps to match the higher resolution of the structural images.

Vasospasm is often localized to specific vascular territories [2], and therefore, it is essential to also monitor CBF at a more granular level throughout the measurement days, both in affected and unaffected regions of the brain. To achieve this, an arterial territories atlas was needed for the purpose of quantifying both the CBF and the variation in CBF within each major arterial territory. The digital 3D brain MRI arterial territories atlas (MNI space) [30], available under the terms of the Creative Commons Attribution-ShareAlike 4.0 International license, was utilized for this purpose. The level 2 version of the atlas (Fig 3) was used, which includes the left and right anterior cerebral artery (ACA), middle cerebral artery (MCA), posterior cerebral artery (PCA), vertebro-basilar artery (VB), and lateral ventricle (LV). The T1w reference (T1 Ref) was registered to the T1w MNI template (MNI152_T1) in two steps. First, a linear registration (using *flirt*) [31, 32] was performed to align the orientation and size of the image, in preparation for the next registration step. Second, a nonlinear registration (using *fnirt*) was conducted using the output from the linear registration and a non-brain-extracted input image. The following step involved reversing this nonlinear mapping (using *invwarp*) and then applying this warp (using *applywarp*) to the atlas in MNI space, thereby transforming the atlas into the reference space (Fig 3).

MATLAB R2020a (MathWorks, Natick, USA) was used for visualization and the remaining analyses. The transformed atlas could be overlaid on each CBF map since they were all in the same reference space. However, because the original ASL acquisition volume was considerably smaller than both the structural T1w image volume and the atlas, the registrations resulted in portions of the CBF maps being zero. Consequently, all values in the CBF map located outside of the imaging area or outside the brain were set to ‘NaN’ and excluded from further calculations. The mean ± standard deviation (SD) CBF was calculated for each territory, along with the spatial coefficient of variation (CoV), defined as the SD divided by the mean, expressed as a percentage. To ensure a smooth map with clear distinctions in CBF, the CBF maps were visualized using the ‘parula’ colormap, modified to display zero values as black. Difference maps (ΔCBF) were also computed between two successive measurements and visualized using a color scheme designed to easily detect changes. In these ΔCBF maps, increased CBF (above 10 ml/min/100g) was shown in magenta, decreased CBF (below -10 ml/min/100g) in yellow, and no larger difference in black.

### Participants

Two healthy female participants, aged 25 (denoted as A) and 35 (denoted as B), were recruited for the measurements on January 31, 2022, and January 30, 2023, respectively. Informed written consent was obtained from both participants in accordance with the Declaration of Helsinki. This study was approved by the local ethics committee (EPN 2018-143/32). Given the potential for brain hemorrhage in any individual, this study has minimal exclusion criteria, provided that patients comply with general MRI safety criteria (e.g., no pacemakers or other MR-unsafe devices). A 50-year-old female patient who had suffered an intracranial hemorrhage after an aneurysm rupture was recruited on March 23, 2023. Informed written consent was obtained from the next of kin (EPN 2021–03527) due to the patient being unconscious. The hemorrhage could be categorized as an SAH, but the accumulated blood was not only limited to the subarachnoid space. This patient was monitored in the NICU for 11 days using the conventional NICU monitoring protocol.

### Evaluation of workflow

Three separate repeated measurements were performed to evaluate the implemented workflow. First, a test-retest experiment was performed to determine the expected within-measurement and the between-measurement variations. Participant A was scanned during two separate sessions (S1 and S2), with a short break in between where the participant stood up before being repositioned in the scanner. The measurement protocol included T1w imaging, followed by two PASL measurements (M1 and M2), and one acquisition of PDw images. In total, two sets of T1w images, four PASL datasets (S1M1, S1M2, S2M1, S2M2), and two sets of PDw images, were acquired. After data processing and analysis, the within-session and between-session differences in ${CBF}_{GM}$ and regional CBFs quantified using the atlas were calculated. In addition, the comparison of two resulting CBF maps was performed by calculating the median difference and the corresponding 1^st^ and 3^rd^ quartiles for each slice. Second, a longitudinal measurement was performed by scanning participant B during six separate sessions over 12 days (Days 1, 3, 5, 8, 9, 12) to simulate a potential monitoring scenario in the NICU. All MR measurements were performed at approximately the same time each morning under consistent conditions. The MR protocol included T1w imaging, PASL imaging, and PDw imaging, as previously described. In addition to the analyses performed in the implemented workflow, the median difference and the corresponding 1^st^ and 3^rd^ quartiles were computed per slice for the five ΔCBF maps. Third, to demonstrate clinical proof of concept, the SAH patient was scanned on two separate occasions (days 6 and 8 after the primary insult) while being monitored in the NICU. During the MR scans, the patient had a ventricular drain and a microdialysis catheter implanted in the brain. PASL, PDw, and T1w images were acquired on both measurement sessions.

## Results

### Test-retest measurement

**Fig 4:**
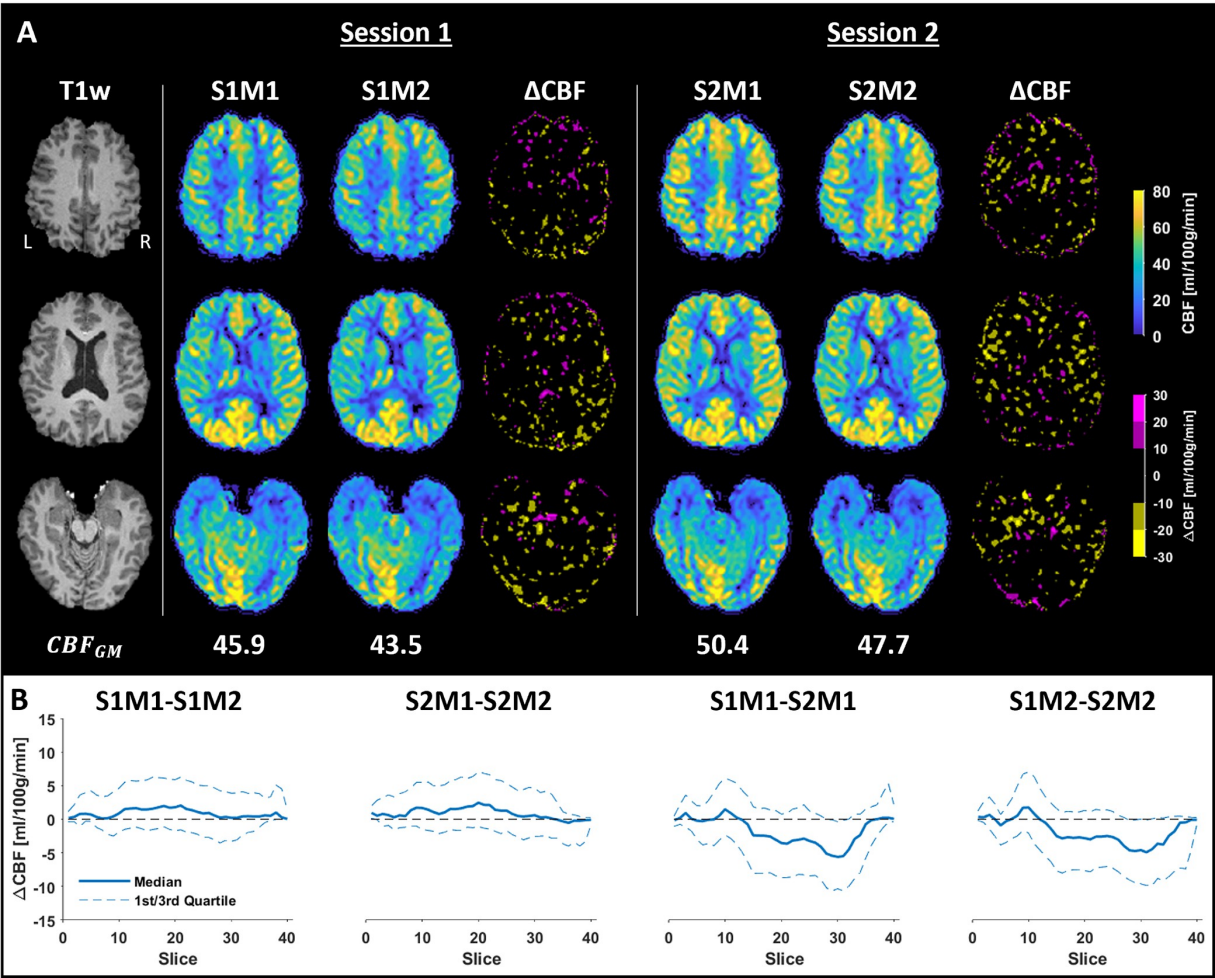
Results from the test-retest experiment. (**A**) The resulting CBF maps from sessions 1 and 2 are shown in three different sections, along with the corresponding ΔCBF maps. In the ΔCBF maps, increased CBF is indicated in magenta, decreased CBF in yellow, and no larger difference in black. The resulting mean ${CBF}_{GM}$ is displayed below each map. (**B**) The median ΔCBF, along with the corresponding 1^st^ and 3^rd^ quartiles, was calculated for each slice. The first two plots investigated within-session differences, while the two last plots examined between-session differences. S1M1 indicates that data from Session 1, Measurement 1 was used in the calculation.

**Fig 5:**
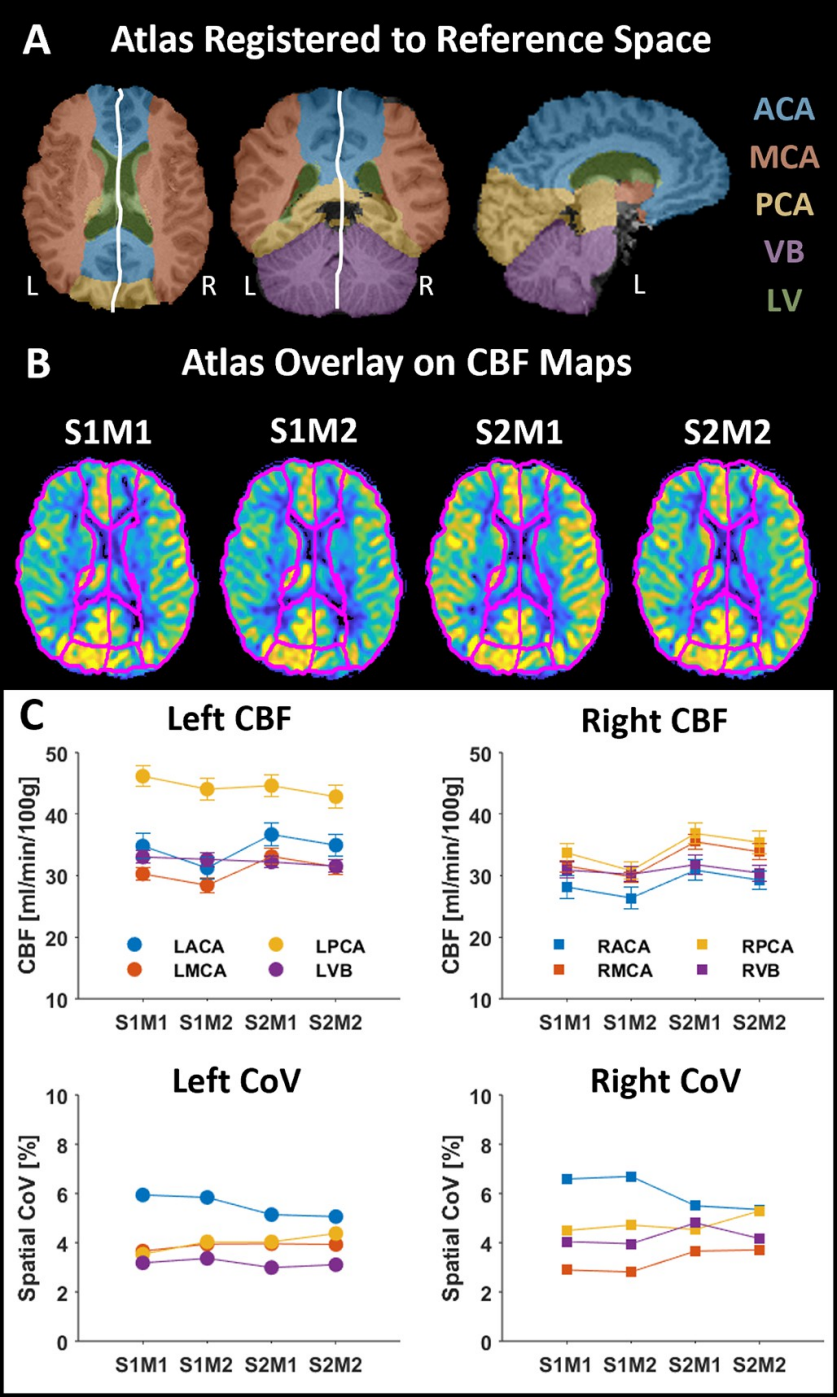
Quantification of the test-retest data. (**A**) The atlas was registered to T1 reference space. The white line separates the left (L) and right (R) arterial territories. (**B**) The registered atlas was overlaid on the CBF maps from the different session, showing a consistent fit among the maps. (**C**) The CBF and spatial CoV were calculated for each arterial territory using the fit in (B). No clear trends were observed in the quantified test-retest data.

Figs 4 and 5 illustrate the registered absolute CBF maps and the results from the atlas quantification for the test-retest measurement. The maps acquired within the same session showed very similar appearances, as indicated by the small changes in the ΔCBF maps (Fig 4). The obtained ${CBF}_{GM}$ values suggested an expected within-session difference of 2.6 ml/min/100g and a between-session difference of 4.4 ml/min/100g. This lower expected within-session difference was also apparent, with few regional exceptions, when investigating the median ΔCBF per slice (Fig 4B) and the quantified CBF values in the flow territories (Fig 5). Another observation was that the test measurements generated higher CBF values than the retest measurements, evident both globally and regionally in the maps. While there were regional differences in the mean CBF between the test and retest measurements, no clear trends were observed in the standard deviation or spatial CoV (Fig 5C). In addition, the LPCA region exhibited a higher CBF than the corresponding territory on the right side, and the highest spatial CoV was observed in the ACA territories.

### Longitudinal measurement

**Fig 6:**
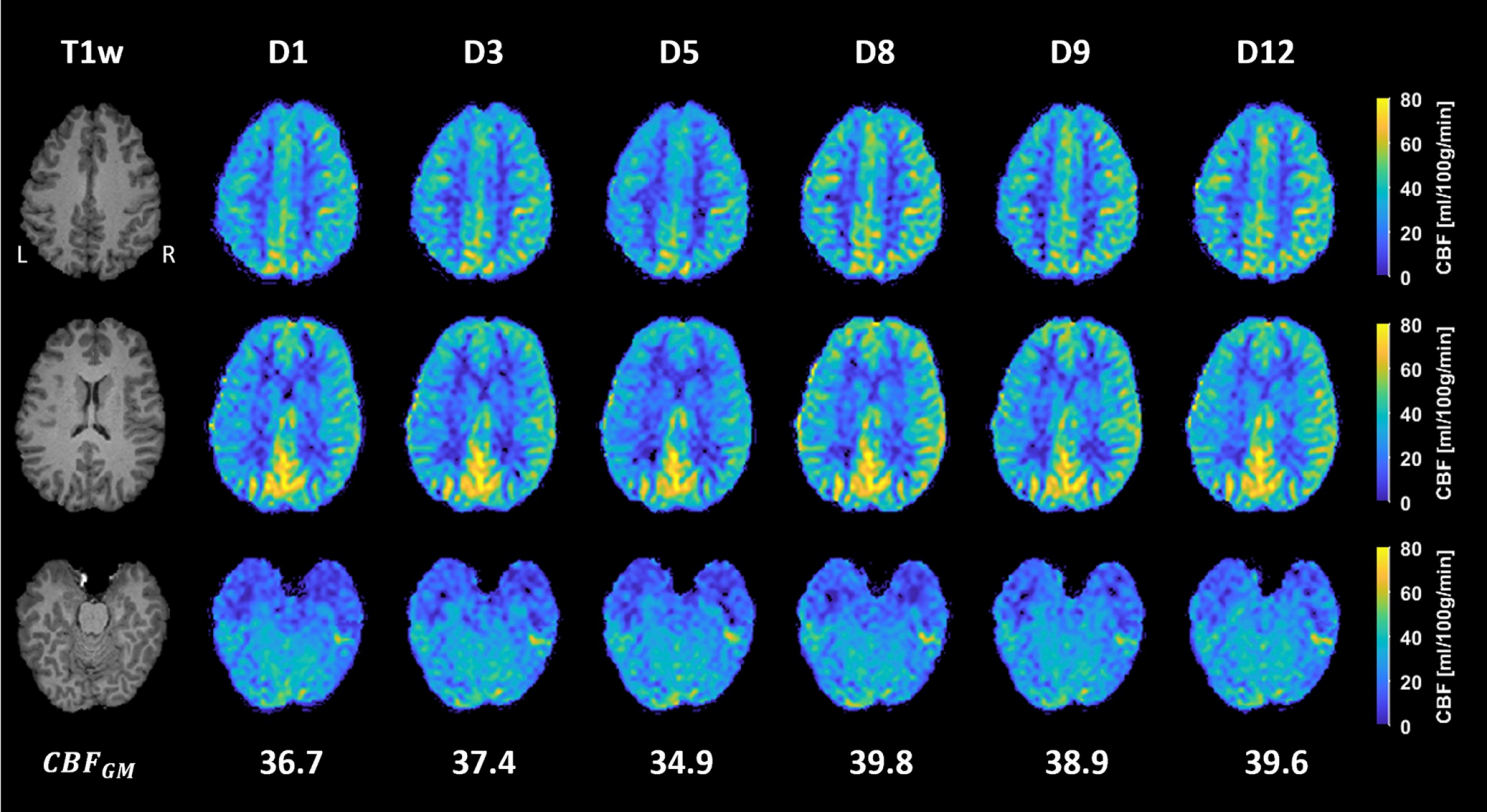
Results from the longitudinal measurement. Structural T1w images are shown on the left, and the resulting absolute CBF maps are displayed for each measurement day (D1 indicates data from day 1). Three representative slices were chosen from the top, middle, and bottom part of the brain. The resulting mean ${CBF}_{GM}$, calculated from the whole CBF map, is shown for each measurement. The appearance of the maps was consistent throughout the 12 days, as expected for a healthy volunteer.

**Fig 7:**
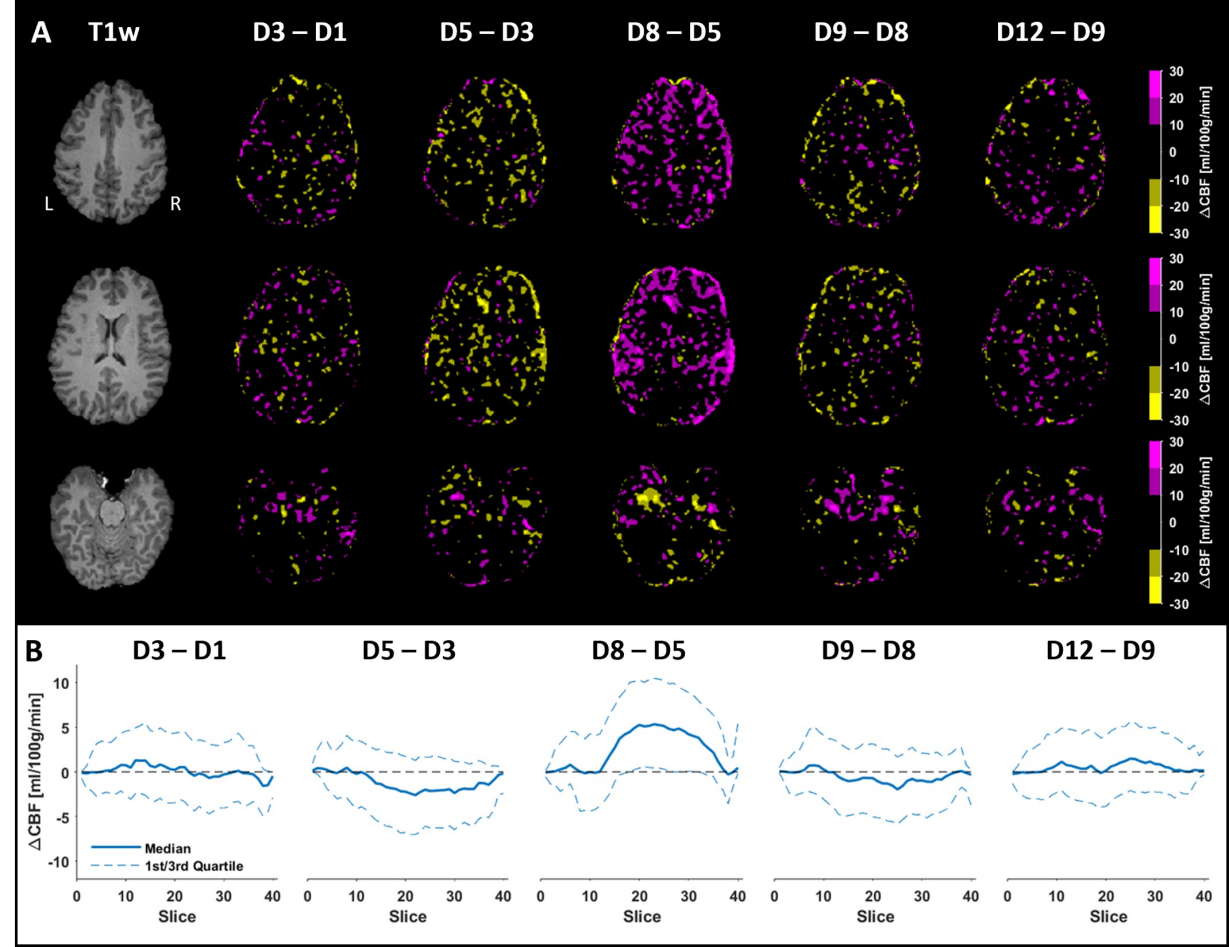
ΔCBF maps computed between CBF maps from two consecutive measurements. (**A**) Structural T1w images are shown to the left and the resulting ΔCBF maps are displayed for each of the five cases (D3-D1 indicates ΔCBF maps calculated between Day 3 and Day 1). The top, middle, and bottom panels correspond to the same representative slices as in Fig 6. In the ΔCBF maps, increased CBF is indicated in magenta, decreased CBF in yellow, and no larger difference in black. (**B**) Median change in ΔCBF, along with the corresponding 1^st^ and 3^rd^ quartiles, was calculated per slice. The changes in CBF were mainly centered around zero, with exception between days 5 and 8, where an increase in CBF was observed.

**Fig 8:**
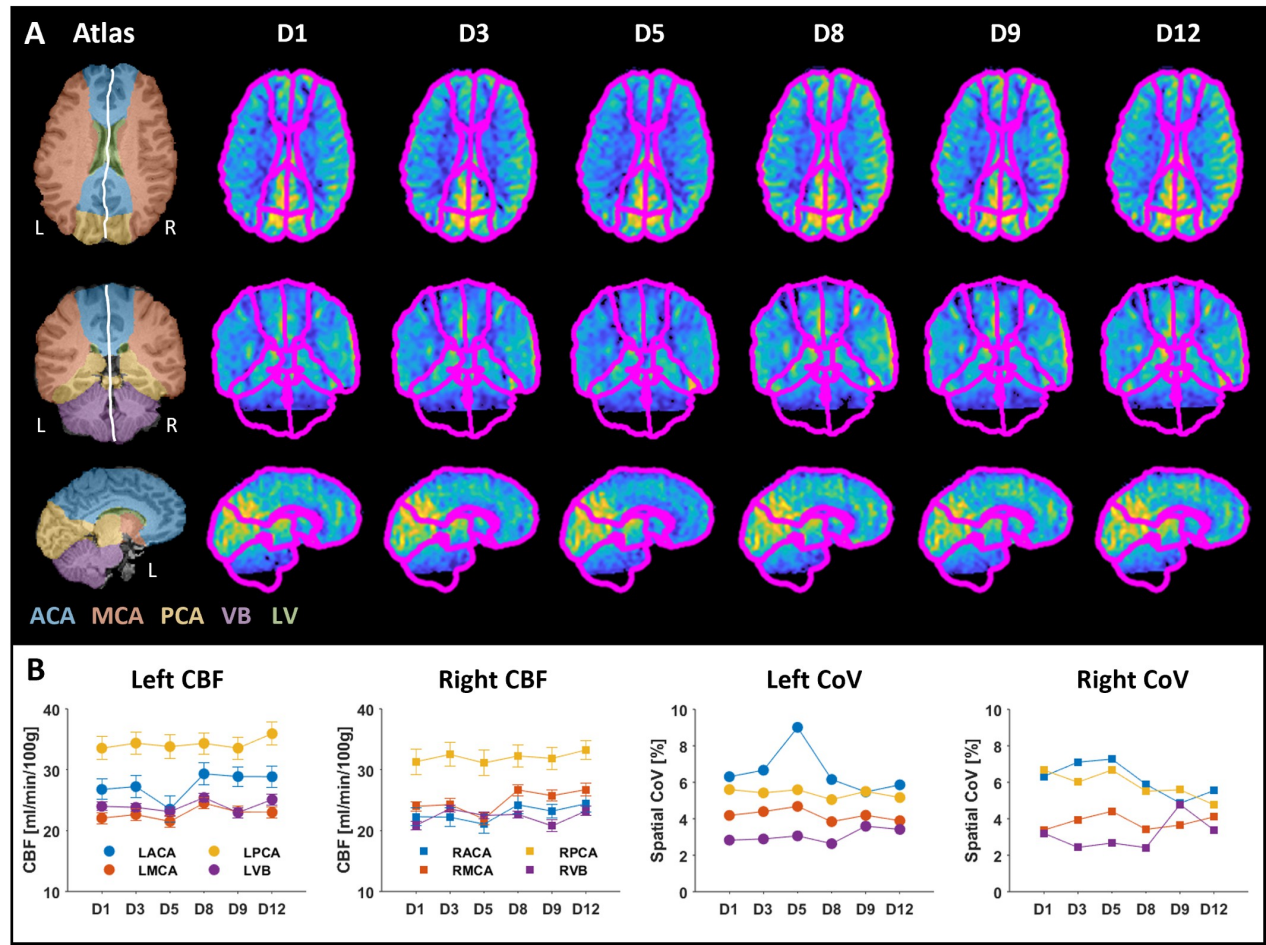
Quantification using the atlas. (**A**) The registered atlas was overlaid on the CBF maps from the longitudinal measurement and is plotted in three different orientations. (**B**) The resulting CBF (mean ± SD) and spatial CoV were calculated for each flow territory across the measurement days. The resulting values were mainly stable throughout the days, with day 5 being an exception. Note that the black areas in the maps are outside the imaging volume, and therefore, not included in the calculations. The territories and their corresponding CBF values are consistently color-coded throughout this figure.

Fig 6 illustrates the processed and registered absolute CBF maps computed from the six acquisitions performed during the longitudinal MR measurement. Minor intensity differences can be observed between the maps, which also were reflected in the corresponding mean ${CBF}_{GM}$ values. Fig 7 shows the calculated ΔCBF maps, where a mix of increased and decreased CBFs was observed, with the largest differences mainly concentrated at the edges. Notably, a lower CBF was obtained on day 5, as evident by a lower resulting ${CBF}_{GM}$ (Fig 6), an almost entirely magenta ΔCBF map (Fig 7A), and a predominantly positive median ΔCBF per slice (Fig 7B). Otherwise, the changes in CBF were mostly centered around zero. Fig 8 displays the registered atlas overlaid on the resulting CBF maps and the corresponding quantified CBF values in different flow territories. The results from day 5 are particularly distinct, especially in the LACA territory, and to a lesser extent in the RACA and MCA territories. The spatial CoV was also higher in these territories, indicating a greater spread in CBF. The highest CBF was observed in the PCA regions, and a lower CBF for day 5 was not detected here. It is also important to note that the CBF map does not cover the entire atlas, and areas outside the acquired region were not included in the calculations (Fig 8).

### Clinical implementation

**Fig 9:**
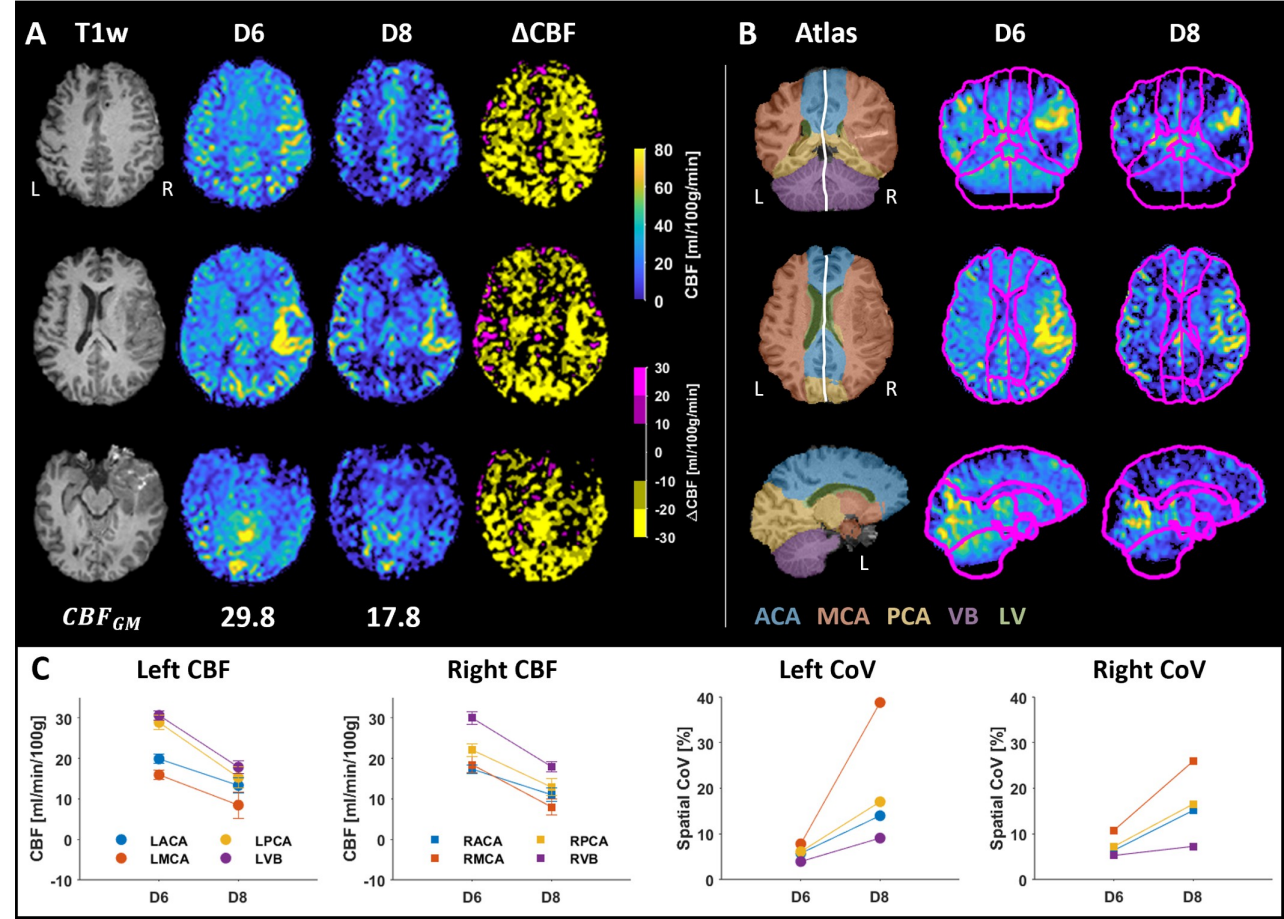
Results from the patient measurements. (**A**) The resulting CBF maps and ΔCBF map are shown in three different sections, computed from patient measurements performed on day 6 (D6) and day 8 (D8) while monitored in the NICU. (**B**) The registered atlas is overlaid on the CBF maps, showing the different flow territories. (**C**) Quantified CBF and spatial CoV in the different arterial territories. On day 8, the flow changed drastically, as indicated by all quantified parameters. Notably, a lower global CBF was observed, which appeared more dispersed, as indicated by the higher spatial CoV.

Fig 9 illustrates the resulting absolute CBF maps, ΔCBF map, and quantification of regional CBF in the flow territories for the patient measurements. The CBF maps reveal areas with no perfusion (black regions), hyperperfused areas around the injury, and some hyperperfused regions on the undamaged left side. However, the absolute CBF maps clearly shows significant decrease in CBF between the two measurement days, reflected by the predominantly yellow ΔCBF map and lower ${CBF}_{GM}$ obtained on day 8. The reduction in CBF was also observed regionally across all flow territories. A much larger spatial CoV was noted on day 8, evident from the more dispersed appearance of the CBF map. The largest changes were primarily observed around the injury on the right side, but similar changes were also seen on the left side.

## Discussion

The implemented workflow includes the acquisition, processing, quantification, and visualization of repeated ASL MRI measurements within the NICU. This workflow was evaluated through test-retest and longitudinal measurements on healthy participants, and practically tested on an SAH patient under NICU monitoring. Both the longitudinal and clinical proof-of-concept measurement demonstrated that repeated ASL measurements are feasible for routine clinical monitoring of NICU patients.

### Generation of absolute CBF maps

The choice of processing steps and calibration method has a significant impact on the resulting CBF map [33]. In this work, the two main aspects influenced the choice of method: the desire to have the CBF maps in absolute units of ml/min/100g, and enabling day-to-day comparison between repeated measurements where appropriate. The CBF maps were calibrated to absolute units using a voxelwise approach, which has the advantage of performing an intrinsic correction of the bias field but might introduce noise in the map. Using a voxelwise approach and considering partial volume estimates is especially important in populations with atrophy [34] or when analyzing CBF maps in native space, as done in this work when calculating ${CBF}_{GM}$. In addition, to retain information about the distribution of CBF in the brain, the processing did not include any smoothing or edge correction of the measurement data. Some edge effects were observed in the resulting CBF maps for all healthy participant measurements, which could potentially be corrected by erosion and extrapolation of the calibration image [25]. Motion correction was another chosen processing step, crucial when scanning healthy participants or non-sedated patients. During the NICU monitoring period, patients are often sedated, which would also be the case during MRI acquisitions, reducing the risk of movement during acquisitions. However, for consistency, the same processing and analysis steps were performed on both healthy participants and patients, regardless of sedation status.

When having multiple data from the same participant/patient, it is important to register the absolute maps to the same space to enable day-to-day comparison of the measurements. Registering all maps acquired from a participant to one of the participant’s structural T1w images provides a better individual reference space for the analysis compared to transforming all CBF maps to the same MNI space. Using MNI space may be a great alternative if CBF maps from different participants need to be compared. It is important to note that these registrations to a reference space may introduce artifacts in the ΔCBF maps if the registration is poor.

### Quantification of absolute CBF maps

The results indicated that participant A had an approximate ${CBF}_{GM}$ of 47 ml/min/100g, while participant B had a ${CBF}_{GM}$ of 38 ml/min/100g. There is evidence that the CBF differs between individuals and that there are both sex- and age-related differences [35]. The conflicting reports on the healthy CBF mainly stem from using different measurement tools and analysis techniques, but a reported value around 50 ml/min/100g in healthy brain was common [36]. Therefore, it is difficult to determine the accuracy of the absolute CBF values in this work. However, the absolute CBF values were relatively stable throughout the repeated measurements for both participant A and B, indicating that larger fluctuations in individual CBF could be detected using the same workflow for all data. Furthermore, the test-retest measurement indicated that a 4 ml/min/100g difference in CBF could be expected between two measurement sessions. The constantly higher CBF obtained for the first measurement in each session was an interesting observation that may be methodological or due to natural causes. In a previous reproducibility study, within-subject test-retest values deviating by less than 20% was considered normal [37]. Another interesting finding in this study was that regardless of quantification method used, the within-session variation was largest in vascular regions, while the between-session variance was largest in GM. These results highlight the importance of considering expected methodological and natural variations when interpreting clinical results.

The variation in resulting CBFs obtained from the longitudinal measurement was minimal, and the CBF remained stable throughout the measurements, with one exception on day 5. The measurements were acquired at approximately the same time each day to minimize variation. However, no control was made for other confounders, such as caffeine intake, which has shown to affect CBF [38].

The same patterns were seen when using the flow territory atlas for quantification of regional CBF. There was a higher CBF in the test measurements compared to the retest measurements, and a lower CBF was noted on day 5 in the longitudinal measurement. However, using the atlas, it was possible to observe that the CBF only changed in the ACA and MCA territories, while remaining stable in the posterior region. Another interesting observation was that the CBF was highest in the posterior region, while the variation was highest in the anterior region of the brain. These observations may be due to methodological reasons, but the higher CBF in the posterior region could be explained by the participants not keeping their eyes closed during the acquisition [39].

The resulting ${CBF}_{GM}$ and the CBF calculated using the atlas were somewhat correlated in the measurements, despite the very different approaches used for their calculation. For example, the ${CBF}_{GM}$ may be inaccurately calculated due to the challenge of correctly characterizing tissue in the segmentation step. In addition, the flow territories in the atlas contain a mixture of tissues, which lowers the achieved CBF because of the lower CBF in white matter (around 20 ml/min/100g) compared to GM [40].

### Patient measurements

Both the clinical availability and demand for ASL MRI have increased in recent years, including applications in acute ischemic stroke, brain tumors, neurodegenerative diseases, and seizures [12, 24]. However, only one previous study could be found using repeated ASL measurements of CBF in the context of vasospasm. In this case report by Romano et al., (2019), a patient diagnosed with contrast-induced encephalopathy after intracranial stent embolization was scanned using ASL three times over three days. The approach was promising for diagnosing vasospasm using ASL because conventional imaging techniques were unsuccessful [41]. This result holds promise for our unique clinical application of NICU monitoring, as we aim for early detection of vasospasm and other changes in CBF to prevent secondary brain injuries in patients after suffering a SAH.

As previously stated, the longitudinal measurement of the healthy participant aimed to mimic a potential monitoring scenario in the NICU. When the analysis workflow was tested on repeated patient data, the results were not as easily interpreted. A global and drastically reduced CBF was observed between the two measurement days, indicated by ${CBF}_{GM}$ dropping from 29.8 to 17.8 ml/min/100g, which was also seen on a regional level in the flow territories. Previous experiments have shown that a reduction of normal CBF below 50% induces immediate loss of neural function in primates, and that a loss of function is observed around 20 ml/min/100g in humans [36]. However, even though the absolute values were below the threshold for ischemia, this patient was considered stable during the MR measurement, as indicated by other monitoring devices (monitoring pulse, blood pressure, respiratory rate, and intracranial pressure). It is important to note that internal CBF preservation is crucial for the patient, but many factors such as temperature, heart rate, blood pressure, and drugs can change the patient’s CBF. Monitor devices showed that the patient’s heart rate increased from 65 (day 6) to 90 (day 8) between the two measurements, which may also be a factor in this result. Alternatively, the arterial transit time might have changed for the patient, causing the labeled blood not to reach the imaging area in time for the acquisition. Despite this possibility, it is still relevant that there was a change for the patient during this monitoring period.

Another important aspect of patient measurements was the severity of the hemorrhage. Potential changes in CBF may vary between patients and depend on the how and where the blood from the hemorrhage is spread throughout the brain. Therefore, it is crucial to conduct individual investigations and consider both global and regional changes in CBF during patient measurements.

### Limitations and future work

The advantages of having an MRI scanner located in the NICU department are numerous, including minimal risks associated with patient transportation. However, performing these repeated MR measurements daily is resource-intensive and may not be necessary for optimal patient care. Other limiting factors include MR safety considerations for other monitoring devices used and the patient’s medical history. In addition, when using the PASL technique, the sensitivity of the timings of the labeling is a known limitation [19], which could be improved by choosing another labeling approach. However, this is a clinical scanner used in the clinical environment, with only PASL currently approved for clinical use. The PASL sequence is easy to implement, conceptually more straightforward than CASL, and less affected by magnetization transfer effects due to the shorter labeling pulses [34].

This study included only two healthy participants, which limits the ability to draw strong conclusions regarding within-measurement and between-measurement variations. However, given that the clinical MR scanner is in the NICU, the opportunity to perform numerous measurements outside of patient care is limited. Additionally, the logistics of obtaining research participants for repeated measurements over a 14-day period, as well as ensuring scanner availability, present significant challenges.

It is also important to remember that the MRI measurements are snapshot estimates reflecting the CBF at the time of the scan. Considering both the patient measurements performed in this work and previous evidence of CBF being influenced by external factors, it is interesting to measure CBF continuously and globally in the brain. This scenario could only be achieved by integrating different techniques to measure CBF in the clinical NICU monitoring protocol. Therefore, future work includes 2D flow (NOVA flow, VasSol Inc., USA) measurements to determine the total inflow of blood to the brain and investigate how this blood is distributed using ASL. In addition, comparing the global CBF obtained using ASL to the CBF achieved from local laser Doppler flowmetry recordings [42].

## Conclusion

This work aimed to implement and test a workflow for the acquisition and analysis of repeated MRI measurements of CBF in the clinical NICU setting. Measurements on healthy volunteers demonstrated the feasibility of applying this workflow to patients in the NICU who are being monitored after suffering cerebral hemorrhages to prevent secondary insults. Although clinical data proved more challenging to interpret than data from healthy participants, the developed workflow, including the analysis and visualization of CBF maps, aided in the interpretation. This workflow shows promise in detecting altered CBF across different measurement days, potentially complementing conventional NICU monitoring techniques by providing additional global and regional CBF information.
